# Patients with complex chronic conditions: Health care use and clinical events associated with access to a patient portal

**DOI:** 10.1371/journal.pone.0217636

**Published:** 2019-06-19

**Authors:** Mary E. Reed, Jie Huang, Richard J. Brand, Romain Neugebauer, Ilana Graetz, John Hsu, Dustin W. Ballard, Richard Grant

**Affiliations:** 1 Division of Research, Kaiser Permanente, Oakland, California, United States of America; 2 University of Tennessee Health Science Center, Memphis, Tennessee, United States of America; 3 Department of Health Care Policy, Morgan Institute for Health Policy, Massachusetts General Hospital, Boston, Massachusetts, United States of America; 4 Department of Health Care Policy, Harvard Medical School, Boston, Massachusetts, United States of America; 5 Kaiser Permanente San Rafael Medical Center, Kaiser Permanente, San Rafael, California, United States of America; University of Oxford, UNITED KINGDOM

## Abstract

**Background:**

For patients with diabetes, many with multiple complex chronic conditions, using a patient portal can support self-management and coordination of health care services, and may impact the frequency of in-person health care visits.

**Objective:**

To examine the impact of portal access on the number of outpatient visits, emergency visits, and preventable hospitalizations.

**Design:**

Observational study comparing patients’ visit rates with and without portal access, using marginal structural modeling with inverse probability weighting estimates to account for potential bias due to confounding and attrition.

**Setting:**

Large integrated delivery system which implemented a patient portal (2006–2007).

**Patients:**

We examined 165,447 patients with diabetes defined using clinical registries. Our study included both patients with diabetes-only and patients with multiple complex chronic conditions (diabetes plus asthma, congestive artery disease, congestive heart failure, or hypertension).

**Measurements:**

We examined rates of outpatient office visits, emergency room visits, and preventable hospitalizations (for ambulatory care sensitive conditions).

**Results:**

Access to a patient portal was associated with significantly higher rates of outpatient office visits, in both patients with diabetes only and in patients with multiple complex conditions (p<0.05). In patients with multiple complex chronic conditions, portal use was also associated with significantly fewer emergency room visits (3.9 fewer per 1,000 patients per month, p<0.05) and preventable hospital stays (0.8 fewer per 1,000 patients per month, p<0.05). In patients with only diabetes, the results were directionally consistent but not statistically significantly associated with emergency room visits and preventable hospital stays.

**Limitations:**

Observational study in an integrated delivery system.

**Conclusion:**

Access to a patient portal can increase engagement in outpatient visits, potentially addressing unmet clinical needs, and reduce downstream health events that lead to emergency and hospital care, particularly among patients with multiple complex conditions.

## Introduction

Diabetes and other chronic diseases account for significant levels of morbidity and mortality in the United States, with an increasing proportion of patients living with multiple complex chronic conditions [1–3]. Multiple chronic conditions are associated with coordination challenges for both patients and health care providers, often across several clinicians and sites of care [1, 4–7]. This complexity can lead to less than optimal treatment, potentially redundant care, and preventable acute services [1, 7–24]. Patient portal tools that improve patient access to their own health information, support self-management, and help patients communicate asynchronously with providers offer an additional mechanism for delivering high-quality guideline-recommended care that can improve patient health [25, 26].

Electronic patient portals, linked to the patient’s clinical electronic health record (EHR), offer patients secure access to their own medical information and the ability to manage several aspects of their health care at any time of day or night through interactive tools, including lab result review, visit summaries, secure messages to providers, and medication refill orders. In some situations, this convenience may allow patients to communicate with health care providers while avoiding traveling to medical facilities and pharmacies, and without requiring time-off from work or care-giving[27–30]. Patient portal access can potentially improve patient engagement and shift the way that health care is delivered, but prior evidence is mixed about the overall portal-related impacts on in-person health care use [28, 31].Healthcare quality improvements have been associated with specific portal components such as patient-provider secure messages use or patient access to lab results [26, 28, 31–33]. However, to our knowledge, ours is the first study to specifically examine overall portal impacts in a population of patients with multiple complex conditions.

For patients with diabetes, many also with other complex chronic conditions, who are likely juggling multiple medications, lab tests, providers, and visits, using a portal to coordinate and manage these may be particularly effective [34]. Our study examines patient portal access in a population with diabetes, including patients with diabetes and additional comorbid conditions, within a large integrated delivery system that first implemented a comprehensive patient portal in late 2005. Our study design takes advantage of historical data from the first two years after the portal was implemented to compare patient health care utilization and events associated with precise timing of each patient’s own individual portal access, with particular attention to patients with multiple complex conditions. We hypothesized that the access to health information, health care self-management tools, and ability to communicate with providers directly through the portal could reduce disease exacerbations and clinical events for patients with multiple chronic conditions who use the portal as measured by emergency room visits and preventable hospitalizations.

## Materials and methods

### Setting

Our study was conducted within the patient population of Kaiser Permanente Northern California (KPNC), an integrated delivery system (IDS) providing comprehensive care for over four million patient members, reflecting the general population in the geographic region. The health system implemented a comprehensive web-based patient portal that integrates all patient in-system health care, including outpatient and inpatient primary care and specialty care, laboratory tests and prescription medications. All delivery-system members can create an online account (register) and use the portal for free to access personal medical information and interactive tools, including viewing lab results and visit summaries, secure email messaging with health care providers, ordering medication refills, and appointment scheduling. While specific updates have been made to the system over time, these core functions have been consistently available to all patients.

Our patient-centered study collaborated with a Patient Partner Panel throughout the research project. Delivery system leaders and clinicians also offered feedback through a Clinician and Delivery System Stakeholder Advisory Group. These panels acted as a sounding board and helped us learn about the patient experience and clinical context for our study and informed the interpretation of study findings. From both groups we understood that impacts on health care visits, especially events like emergency room visits and hospital stays, are key outcomes.

#### Study design

We conducted a retrospective historical observational study using automated data from the portal itself, the electronic health record (EHR), and other delivery system automated databases. Although the portal was initially made broadly available by the health system, since the portal is a patient-facing tool that individual patients must actively login to in order to be directly exposed, our study design exploits variation in timing of a patient’s first registration (index date of exposure) as an individual measure of portal access.

Our goal was to compare patient-time with and without portal access, not the impact of any given specific portal login event. To do this, we defined a patient as having access to the portal once they registered to use it and examined differences in outcomes between time periods during which a given patient had never registered to use the portal (patient has ‘no portal access’) and outcomes after the patient had registered to start using the portal (patient has ‘portal access’). In other words, this is an intent-to-treat analysis that assumes once a patient registered to access the portal, he or she would have access to use the portal thereafter.

Since patient characteristics could influence both portal use and the subsequent outcomes, we used analysis methods with multiple dynamic patient covariates in an effort to manage potential confounding over time [35]. We hypothesized that many patient characteristics would directly influence initial portal access, including socio-demographic characteristics and clinical need close to the time that they first become users of the portal. Prior studies have documented a short-term uptick in office visits before a given patient portal use [31, 36, 37]. To account for this, we used precise time-changing clinical measures to capture short term changes in clinical need (visits, medications, and medical tests) preceding portal access. In this observational study, we chose marginal structural models (MSM) fitted by dynamic monthly inverse probability weighting (IPW) estimates to account for potential bias due to confounding and attrition when evaluating the effect of portal access on health events.

The study design and detailed analysis plan were designed a priori and align with PCORI Methodology Standards. The study activities were reviewed and approved by the Institutional Review Board of the Kaiser Foundation Research Institute, which waived the requirement for informed consent in this data-only study.

### Study period and population

Our study population included all patients in the clinical chronic conditions registry for diabetes (see supplemental material), defining a subset of patients with complex chronic conditions if they had one or more other chronic conditions, identified using ICD-9/10 based clinical registries for asthma, congestive heart failure, coronary artery disease, and hypertension [38]. The registries, defined for active clinical care and quality measurement unrelated to this research project, have also been used in prior research.

Our study examined outcome data from January 2006 to December 2007, the first two calendar years after portal initial implementation in late 2005. While this time period is historical, using this timeframe directly after portal implementation allowed us to better capture changes due to portal implementation rather than other external factors. We used monthly longitudinal data to create monthly data for each patient, from January 2006 until the end of the study or the month of disenrollment or death, with information on patient baseline and time-varying covariates, patient portal use status (0 in months before initial portal registration and 1 in and after the first month of registration, with index date defined by the time of registration), and health events.

### Outcome measures

For all patients in our study, we used the clinical patient history data captured in the EHR to extract counts of all outpatient office visits, emergency department (ED) visits, and preventable hospitalizations defined by admissions for ambulatory care sensitive conditions (ACSCs) [39], including external claims. We calculated monthly counts of these events separately for each patient (for each month in the study period). Within the transition month when a patient first became a user of the portal, we counted the number of events after the date of registration and then adjusted for the partial month by dividing by the total remaining days in the month and multiplying by 30.

### Statistical analysis

We used a marginal structure model with inverse weighting (stabilized weights) estimation, to account each month for differences in users and non-users of the portal, in evaluating the impact of patient portal access on health events. First, we created a data file with each person-month as a separate record, sorted by patient and month. Second, we ran three separate pooled logistic regression models to predict monthly probabilities of portal registration, censoring by disenrollment, and censoring by death using both baseline and time-varying covariates[40] for the denominator of the stabilized weights. Predictors in logistic regression for denominator included age, sex, race/ethnicity, neighborhood SES (based on 2000 census measures for the census block group of the patient’s residential address), prior year’s rate of chronic disease prescription drug adherence (proportion of days covered), prior year health status (number of hierarchical condition categories) [41], neighborhood internet access level (Federal Communications Commission’s percentage of households with residential high-speed internet access in 2008), and prior health care utilization rates (office visit, phone visit, ED visit, hospitalization) both short term (prior 30 days) and longer term (prior 2–6 months), and the calendar month. Similarly we ran three separate pooled logistic regression models for the numerator with calendar month as the predictor. Third, we calculated the product of the probability of a patient getting the exposure in the given month (probability of not having portal access if the patient did not have access yet to the portal or probability of having access in the given month if the patient had access to the portal), probability of remaining enrolled in the integrated delivery system, and probability of remaining alive in the given month for both numerator and denominator. Then, within each patient, we calculated the final numerator by multiplying the numerator of current month with the numerators from all prior months. Similarly, we calculated the final denominator by multiplying the denominator of current month with all the denominators from prior months. Then we calculated stabilized weights by dividing the final numerator by the final denominator for each person-month. We truncated the stabilized weights at the 99^th^ percentile (1.88, see supplement for more detail).

Finally, we used PROC GENMOD in SAS to fit the weighted linear regression model with an indicator of being ever registered for the portal, adjusting for time trend (indicator variable for study month) to estimate the impact of portal access and its robust standard error. We repeated the analyses in two subgroups stratified by chronic condition complexity: patients with diabetes only, and patients with complex chronic conditions. We examined the impact in these two subgroups because of an a priori hypothesis that patients with more complex conditions may potentially benefit more from any portal use. In a sensitivity analysis to examine the performance of the inverse probability weights we used weights from logistic regression with several interaction terms in addition to the main effects, and a machine learning approach known as Super Learning [42, 43] (a data adaptive estimation approach based on cross-validation and predictors defined by logistic regression and polychotomous regression, see S1 Fig, S1 Table, and S2 Table) and found results to be comparable across all sensitivity analyses. All main analyses were completed using SAS 9.3 (SAS Institute Inc., Cary, NC), with Super Learning analysis conducted with statistical software R (version 2.15.2).

## Results

Among all study patients with diabetes (N = 165,477), 77.4% had multiple chronic conditions (diabetes plus one or more other conditions, Table 1). During the study period 22.3% of all study patients started to use the patient portal (see supplement).

**Table 1:** Baseline patient characteristics of patients with diabetes (N = 165,477).

			Portal access in study period	
Baseline Characteristics		All Patients	Portal Access	No Access
Age	<65	55.6%	65.2%	52.9%
	65–74	24.2%	21.3%	25.0%
	75+	20.2%	13.5%	22.2%
Gender	Male	51.9%	52.3%	51.8%
Race/ethnicity	White	48.6%	60.9%	45.0%
	Black	11.5%	8.0%	12.5%
	Hispanic	20.6%	13.1%	22.7%
	Asian	17.6%	16.6%	17.9%
Neighborhood SES*	Low	24.1%	16.9%	26.2%
Neighborhood internet access^‡^	<40%	13.2%	9.5%	14.3%
	40-<60%	21.8%	19.2%	22.6%
	60-<80%	33.9%	35.0%	33.6%
	80%+	21.5%	27.0%	19.9%
Medication adherence^†^	Yes	73.7%	77.3%	72.7%
Multiple chronic conditions**	Yes	77.4%	76.3%	77.7%

Portal access status in this table is defined based on registering to use the portal at any time during the longitudinal study period. In analyses of portal access impacts, all patient observation time prior to first portal use during the study period is attributed under their non-user status.

Age reported as of 01/2006

SES = socioeconomic status

*Neighborhood SES based on 2000 census measures for the census block group of the patient’s residential address as of 01/2006 (9.6% unknown due to addresses which cannot be geocoded)

^‡^Neighborhood internet access based on FCC published percentage of households with residential high-speed internet access in 2008 for census tract of the patient’s residential address as of 01/2006 (9.6% unknown due to addresses which cannot be geocoded)

^†^Medication adherence defined for chronic conditions medications as proportion of days covered greater than 80% in 2005

**Chronic conditions defined using clinical registries for asthma, congestive heart failure, coronary artery disease, diabetes, hypertension as of last quarter of 2005

P<0.0001 for comparisons between portal users and portal non-users for all characteristics except gender (p = 0.07).

**Fig 1:**
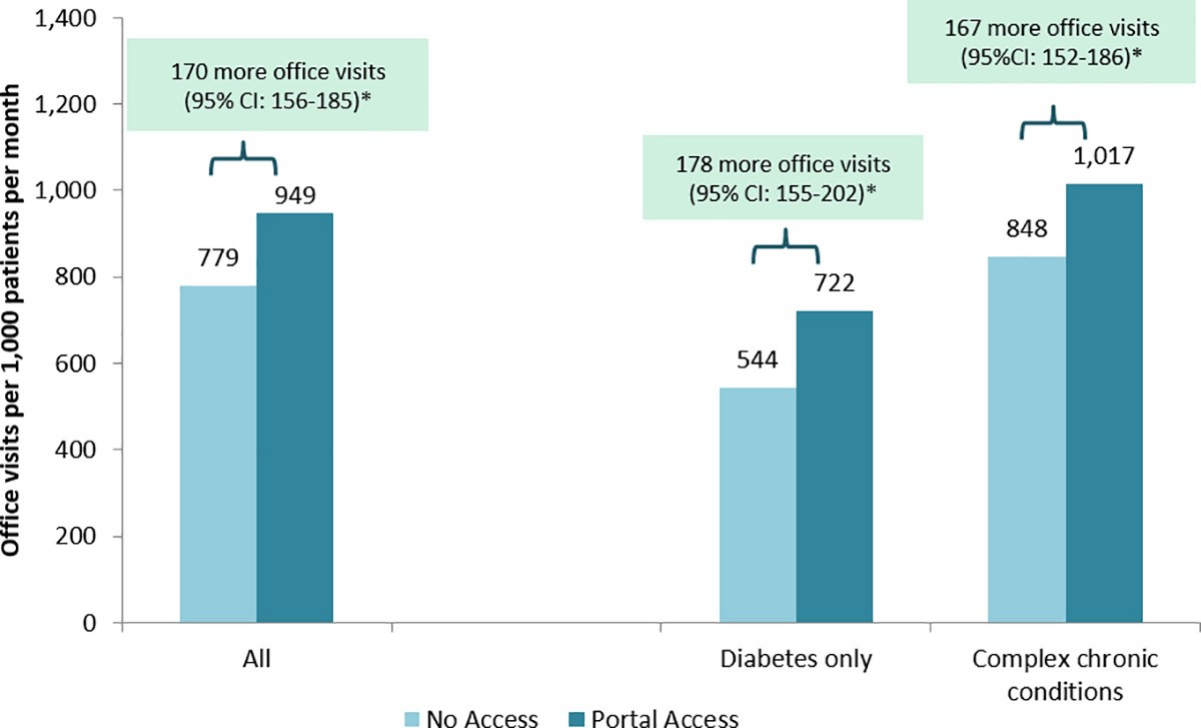
Difference in office visit use associated with portal access in all patients with diabetes and in patients with complex (multiple) chronic conditions. Results based on marginal structural modeling (MSM) with inverse probability weights (IPW) predicted by patient age, gender, race/ethnicity, neighborhood SES, neighborhood internet access, engagement, comorbidity, and office visits, phone visits, ED visits, and hospitalizations in prior 30 days and in prior 2–6 months. Complex chronic conditions defined as diabetes plus one or more other additional conditions among: asthma, coronary artery disease, congestive heart failure, or hypertension. *statistically significant differences (p<0.05) are described with a text box above.

In examining the association between portal use and rates of office visits, ED visits and hospitalizations, we found two distinct patterns. After accounting for patient characteristics and time-varying clinical needs using IPW estimation, access to the portal was associated with significantly more office visits (170 per 1,000 patients per month, p<0.05, Fig 1). This difference was relatively consistent among patients with only diabetes (178 per 1,000 patients per month, p<0.05), and patients with multiple chronic conditions (167 more office visits per 1,000 patients per month, p<0.05).

**Fig 2:**
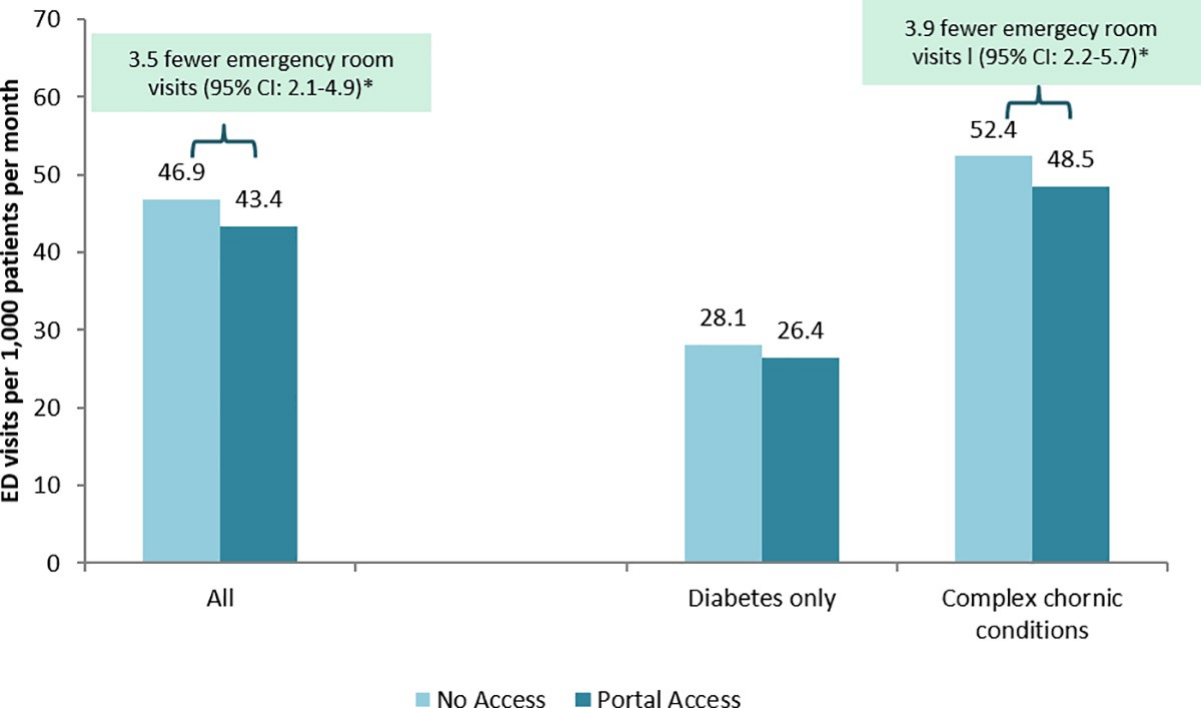
Difference in emergency department visits associated with portal access in all patients with diabetes and in patients with complex (multiple) chronic conditions. Results based on MSM with IPW predicted by patient age, gender, race/ethnicity, neighborhood SES, neighborhood internet access, engagement, comorbidity, and office visits, phone visits, ED visits, and hospitalizations in prior 30 days and in prior 2–6 months. In patients with diabetes only, the difference in ED visits if using the portal was -1.7 (95% CI: -3.9–0.5). *statistically significant differences (p<0.05) are described with a text box above.

**Fig 3:**
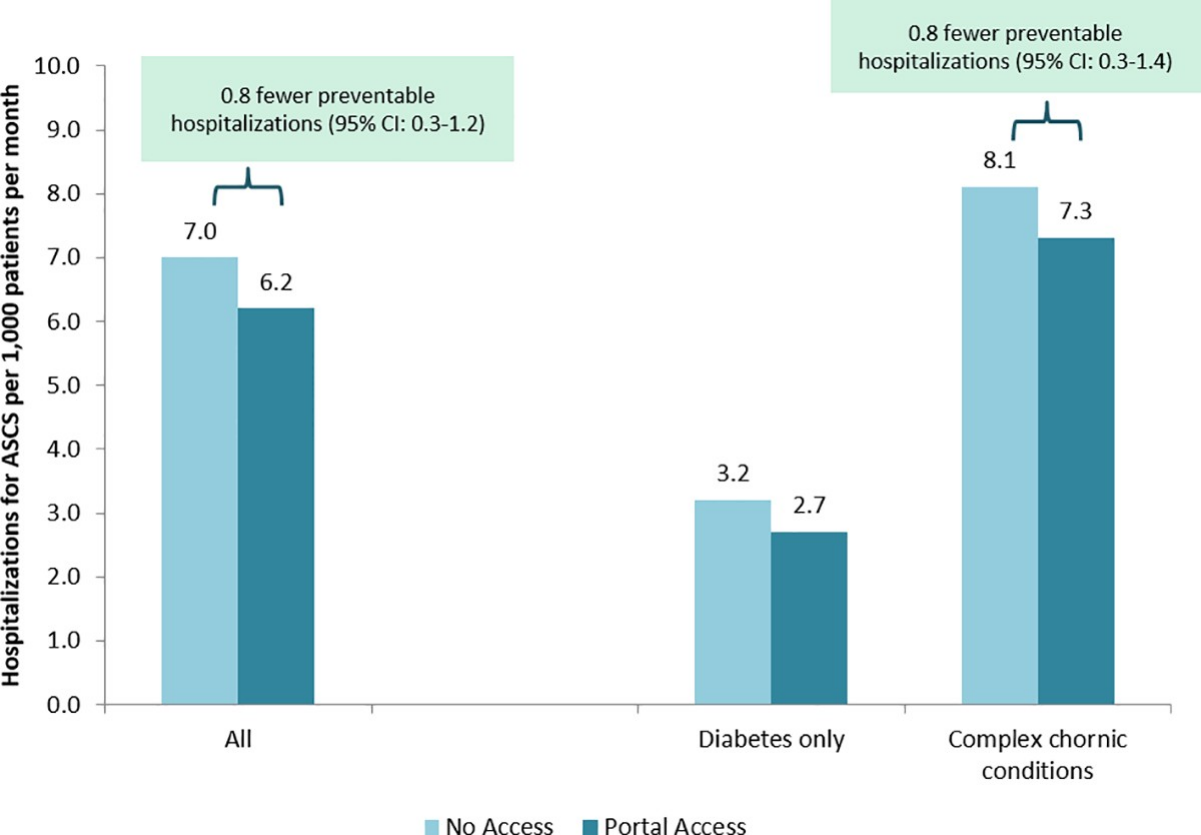
Difference in preventable hospitalizations associated with portal access in all patients with diabetes and in patients with complex (multiple) chronic conditions. Preventable hospitalizations defined as hospitalization for an ambulatory care sensitive condition. Results based on MSM with IPW predicted by patient age, gender, race/ethnicity, neighborhood SES, neighborhood internet access, engagement, comorbidity, and office visits, phone visits, ED visits, and hospitalizations in prior 30 days and in prior 2–6 months. In patients with diabetes only, the difference in hospitalizations visits if using the portal was -0.5 (95% CI: -1.0–0.1). *statistically significant differences (p<0.05) are described in a text box above.

In contrast, portal access was associated with significantly fewer ED visits (3.5 per 1,000 patients per month, p<0.05, Fig 2) and preventable hospital stays (0.8 per 1,000 patients per month, p<0.05) overall. In the population of patients with complex chronic conditions, portal access was associated with significantly fewer ED visits (3.9 per 1,000 patients per month, p<0.05) and preventable hospital stays (0.8 per 1,000 patients per month, p<0.05), as measured by ambulatory care sensitive hospitalizations (Fig 3).

In patients with only diabetes, the results were directionally consistent but not statistically significantly associated with ED visits and preventable hospitalizations (1.7 fewer ED visits per month with portal access, 95% CI -3.9–0.5; 0.5 fewer preventable hospitalizations per month with portal access, 95% CI -1.0–0.1; Figs 2 and 3).

## Discussion

Among patients with diabetes, particularly complex patients with multiple chronic conditions, we examined the association between portal access and office visits and health events as captured by ED visits and hospitalizations. We found that access to a patient portal was associated with engaging in significantly more outpatient office visits. When patients with multiple conditions had access to the portal, however, they were also less likely to have ED visits or preventable hospitalizations, suggesting a reduction in downstream clinical events associated with portal use.

Our study is unique in specifically examining portal impacts in patients with multiple complex chronic conditions. We anticipated that patients with this complexity in health care experience, likely managing multiple providers, visits, medications, and lab monitoring schedules, might have a particular opportunity to benefit from using the portal to facilitate self-management activities. We found that patients with diabetes had more office visits after gaining access to the patient portal. Consistent with prior evidence that higher outpatient healthcare access is associated with lower ED visits [44, 45], we found that the portal-associated increases in office visits were accompanied by a decrease in ED visits. Only patients with multiple conditions experienced statistically significantly fewer health events as measured by emergency department visits and ambulatory-care-sensitive hospital stays, although other patients with diabetes may well benefit as well [46].

In this patient centered project, our patient research partners linked the study’s analytic findings to their own lived experiences in using the portal. For instance, while the statistical analysis found an overall increase in office visit rates associated with portal use, discussions with patients helped clarify that this should be interpreted as an averaged effect, and that individual patient experiences may differ. For example, patient partners related personal situations of directly avoiding an office visit by asking their doctor a question through the portal. In other instances, patient partners reported situations where using the portal to check a lab result led directly to follow-up engagement with their physician through an in-person visit. Similarly, patient partners linked the statistical findings of reductions in emergency visits to personal experiences, for example, in using the portal after regular medical hours to obtain a medication that helped to avoid an ED visit. While individual care-seeking experiences may vary, we found that on average patients experienced fewer ED visits and preventable hospital stays after starting to access the portal.

Our study examines the overall impact of patient portal access on patient health care activity and events. Several other studies have examined specific instances of portal use (a login transaction on a particular date), individual portal tools (such as secure messaging or open notes), or different target populations of patients (such as a general primary care patient population) [26, 28, 31, 33, 36, 37].Some studies, including across different settings within the same integrated delivery system, have reported an association with increases in office visits, and others with decreases, but few have examined downstream clinical events. Our study finds that portal use is associated with a statistically significant reduction in downstream clinical events among patients with higher complexity. These findings of differences in health care utilization and events are consistent with other patient-centered survey findings of portal use being associated with shifts toward greater communication, and patient-reported health improvements [27, 28, 47]. While some patient-reported findings have suggested that portal use may decrease office visits, we interpret our finding of an average increase in office visit activity as likely a combination of individual patient experiences that include both intentional substitution for office visits and increases in office visit engagement. Further research should examine the impact of targeted strategies to educate patients, providers, and health care delivery systems about the potential benefits of and barriers to portal use, including through dissemination of patient-reported experiences.

While prior observational studies have examined impacts of patient portal use on health care utilization, to the best of our knowledge, ours is the first to use a study design that accounts for time-changing clinical needs closely linked to the timing of portal use. Research designs that do not adequately account for time-changing factors close to the time of portal use may mistakenly attribute any increase in use of in-person health care services to using a patient portal, rather than to the new health need. In addition, our findings also incorporate adjustment for a relatively comprehensive list of potential statistical confounders. Thorough adjustment for time-changing covariates is critically important in designing observational studies to assess the impact of patient portal use on health outcomes [34].

There are several limitations to the generalizability of our study findings. Since the study was conducted in a single integrated health delivery system with a comprehensive patient portal, the results may not necessarily generalize directly to other settings. Still, this study setting includes both commercial and public insurance enrollees, represents approximately 33% of the underlying population in areas served, and is highly representative of the surrounding and statewide insured population in race/ethnicity and socio-economic status, with the exception of patients with extremely low income [48, 49]. We do not examine portal uses directly and our intent-to-treat approach examining general portal access may underestimate the impact of specific portal uses and actions. While the time period used in this study is historical, using this timeframe supported a stronger causal study design and analytic methods. Still, in examining a setting that is a technology leader within an integrated setting, the portal tools and functions studied are comparable to current portal implementations, including those used in the current federal Promoting Interoperability program criteria. Also, since the portal offered in this setting was available free of charge to patients, findings may vary when costs, such as for secure messages, are applied. Since our study was focused on patients with diabetes and other chronic conditions captured in available disease registries, the study is limited in its generalizability to other populations without chronic conditions. While our study accounts for many potential confounders and time-changing patient and clinical variables, there may be other patient characteristics associated with using or not using the portal that we could not measure or account for in our analyses. Since this is an observational study, we cannot rule out unmeasured confounding or establish causality.

## Conclusions

Overall, we found that in patients with diabetes within an integrated delivery system offering a comprehensive patient portal, office visit rates were higher when patients had access to a patient portal compared to when they did not, and that portal access was associated with statistically significantly lower rates of ED visits and preventable hospitalizations in patients with complex chronic conditions. Together with patient partners in this patient-centered study, we interpret these findings as a signal that the portal may be helping to increase engagement in outpatient office visits, a preferable setting to potentially address otherwise unmet clinical needs, and thereby reducing downstream health events that lead to emergency and hospital care.

## Supporting information

S1 Fig Number of patients and patients who registered to use the patient portal (cumulative) 2006–2007. (TIF)

S1 Table Sensitivity analyses: Association between patient portal user vs. non-user and clinical outcomes (N = 165,447). (DOCX)

S2 Table Distribution of weights from sensitivity analyses. (DOCX)
