# Supplementary figures and images for: Patients with complex chronic conditions: Health care use and clinical events associated with access to a patient portal
Source: PLoS One. 2019 Jun 19;14(6):e0217636. doi: 10.1371/journal.pone.0217636 (PMC6583978; doi:10.1371/journal.pone.0217636)

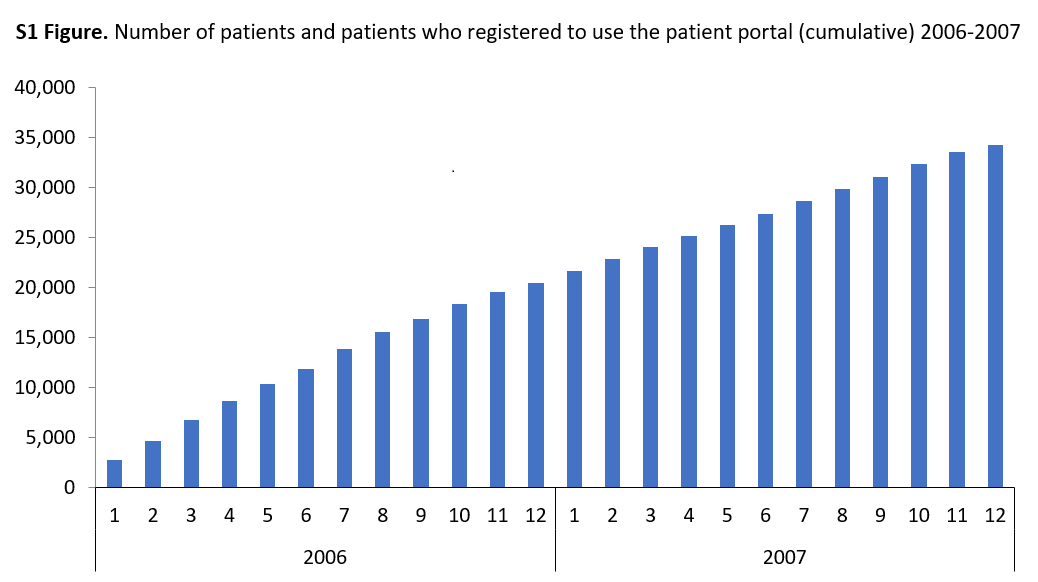

Supplement: S1 Fig — (TIF) [file pone.0217636.s001.tif]
