# Supplementary material for: Patients with complex chronic conditions: Health care use and clinical events associated with access to a patient portal
Source: PLoS One. 2019 Jun 19;14(6):e0217636. doi: 10.1371/journal.pone.0217636 (PMC6583978; doi:10.1371/journal.pone.0217636)
Supplement: S1 Table — (DOCX) [file pone.0217636.s002.docx]

**S1 Table.** Sensitivity analyses: association between patient portal user vs. non-user and clinical outcomes (N=165,447 as of 01/2006)

|  |  | Coef | 95% CI | |
| --- | --- | --- | --- | --- |
| Office visit | Main Analysis | 0.1703 | 0.1557 | 0.1849 |
|  | Sensitivity Analysis 1 | 0.1648 | 0.1495 | 0.1802 |
|  | Sensitivity Analysis 2 | 0.1479 | 0.1334 | 0.1624 |
| ED | Main Analysis | -0.0035 | -0.0049 | -0.0021 |
|  | Sensitivity Analysis 1 | -0.0040 | -0.0054 | -0.0025 |
|  | Sensitivity Analysis 2 | -0.0053 | -0.0066 | -0.0039 |
| IP ACS | Main Analysis | -0.0008 | -0.0012 | -0.0003 |
|  | Sensitivity Analysis 1 | -0.0009 | -0.0013 | -0.0004 |
|  | Sensitivity Analysis 2 | -0.0011 | -0.0016 | -0.0007 |

Model: linear regression adjusted for calendar month -- SAS proc genmod, link=identity, repeated subject=patient mrn, type=ind

Main analysis: presented in manuscript, with weight (truncated at 1.88) from model with main effects only

Sensitivity analysis 1: with weight (truncated at 1.92) from model with main effect and 9 interactions terms: age group*health status, age group*internet access, age group* lowses, race*internet access, race*lowses, event in prior 30 days* event in prior 2-4 months (office, telephone, ED visit and hospitalization respectively)

Sensitivity analysis 2: with weight (truncated at 1.72) from superlearner (glm and polyclass)
