# Supplementary material for: Patients with complex chronic conditions: Health care use and clinical events associated with access to a patient portal
Source: PLoS One. 2019 Jun 19;14(6):e0217636. doi: 10.1371/journal.pone.0217636 (PMC6583978; doi:10.1371/journal.pone.0217636)
Supplement: S2 Table — (DOCX) [file pone.0217636.s003.docx]

**S2 Table.** Distribution of weights from sensitivity analyses

|  | Minimum | Median | 95th Percentile | 99th Percentile | Maximum |
| --- | --- | --- | --- | --- | --- |
| Main Analysis | 0.03 | 0.99 | 1.25 | 1.88 | 8.80E+13 |
| Sensitivity Analysis 1 | 0.01 | 0.99 | 1.24 | 1.92 | 6.60E+13 |
| Sensitivity Analysis 2 | 0.01 | 0.99 | 1.18 | 1.72 | 6.08E+13 |

Note: In the main analysis, we truncated the stabilized weights at the 99^th^ percentile (1.88)
